# Safety and Efficacy of Hypoxia-Inducible Factor-Prolyl Hydroxylase Inhibitors vs. Erythropoietin-Stimulating Agents in Treating Anemia in Renal Patients (With or Without Dialysis): A Meta-Analysis and Systematic Review

**DOI:** 10.7759/cureus.47430

**Published:** 2023-10-21

**Authors:** Nanush Damarlapally, Vijaylaxmi Thimmappa, Hamza Irfan, Muhammad Sikandari, Krupa Madhu, Aayushi Desai, Peddi Pavani, Syeda Zakir, Manvi Gupta, Maha Mushtaq Khosa, Sohny Kotak, Giustino Varrassi, Mahima Khatri, Satesh Kumar

**Affiliations:** 1 Health Sciences and Medicine, Houston Community College, Houston, USA; 2 Prosthodontics, Mariner Dental Laboratory, Houston, USA; 3 Internal Medicine, Shaikh Khalifa Bin Zayed Al-Nahyan Medical and Dental College, Lahore, PAK; 4 Internal Medicine, Shaheed Mohtarma Benazir Bhutto Medical College, Karachi, PAK; 5 Internal Medicine, Gujarat Medical Education and Research Society (GMERS) Medical College, Gandhinagar, Gandhinagar, IND; 6 General Surgery, Kurnool Medical College, Andhra Pradesh, IND; 7 Internal Medicine, Dow University of Health Sciences, Karachi, PAK; 8 Internal Medicine, Subharti Medical College, New Delhi, IND; 9 Internal Medicine, Quetta Institute of Medical Sciences, Quetta, PAK; 10 Pain Medicine, Paolo Procacci Foundation, Rome, ITA; 11 Medicine and Surgery, Dow University of Health Sciences, Karachi, PAK; 12 Medicine and Surgery, Shaheed Mohtarma Benazir Bhutto Medical College, Karachi, PAK

**Keywords:** treatment, anemia, and efficacy, safety, without dialysis, dialysis, kidney, renal, erythropoietin stimulating agents, hypoxic inducible factors prolyl hydroxylase inhibitors

## Abstract

Hypoxia-inducible factor-prolyl hydroxylase domain inhibitors (HIF-PHIs) are a novel group of drugs used to treat renal anemia, but their benefits vary among different trials. Our meta-analysis aims to assess the safety and efficacy of HIF-PHI versus erythropoiesis-stimulating agents (ESA) in managing anemia among patients with chronic kidney disease (CKD), regardless of their dialysis status. PubMed, Embase, and Google Scholar were queried to discover eligible randomized controlled trials (RCTs). To quantify the specific effects of HIF-PHI, we estimated pooled mean differences (MDs) and relative risks (RR) with 95% CIs. Our meta-analysis involved 22,151 CKD patients, with 11,234 receiving HIF-PHI and 10,917 receiving ESA from 19 different RCTs. The HIF-PHI used included roxadustat, daprodustat, and vadadustat. HIF-PHI yielded a slight but significant increase in change in mean hemoglobin (Hb) levels (MD: 0.06, 95% CI (0.00, 0.11); p = 0.03), with the maximum significant increase shown in roxadustat followed by daprodustat as compared to ESA. There was a significant decrease in efficacy outcomes such as change in mean iron (MD: -1.54, 95% CI (-3.01, -0.06); p = 0.04), change in mean hepcidin (MD: -21.04, 95% CI (-28.92, -13.17); p < 0.00001), change in mean ferritin (MD: -16.45, 95% CI (-27.17,-5.73); p = 0.03) with roxadustat showing maximum efficacy followed by daprodustat. As for safety, HIF-PHI showed significantly increased incidence in safety outcomes such as diarrhea (MD: 1.3, 95% CI (1.11, 1.51); p = 0.001), adverse events leading to withdrawal (MD: 2.03, 95% CI (1.5, 2.74), p = 0.00001) among 25 various analyzed outcomes. This meta-analysis indicates that HIF-PHIs present a potentially safer and more effective alternative to ESAs, with increased Hb levels and decreased iron usage in CKD patients without significantly increasing adverse events. Therefore, in these patients, we propose HIF-PHI alongside renal anemia treatment.

## Introduction and background

Anemia is a condition in which the body lacks enough healthy RBCs to carry adequate oxygen to tissues. According to the WHO, anemia is characterized by Hemoglobin (Hb) levels of less than 12.0 g/dL in premenopausal women and less than 13.0 g/dL in men. Chronic kidney disease (CKD)-related anemia is a type of normocytic, normochromic, hypo proliferative anemia. The primary causes of the anemia associated with CKD are decreased erythropoietin (EPO) production and poor iron homeostasis, which result in diminished erythropoiesis [1]. CKD-related anemia results when the glomerular filtration rate (GFR) is less than 60 ml/min, and the kidney is unable to generate enough EPO in response to hypoxia [2]. Anemia is common in CKD patients regardless of the categorization criteria, with over 80% of patients on dialysis. It occurs most frequently in women, older people, and people with co-morbidities such as diabetes, cardiovascular diseases, and systemic illnesses [3]. According to the CDC, the United States has more than 37 million American adults with CKD, with more than one out of every seven people suffering from anemia due to CKD. The currently available treatment option for anemia due to CKD is erythropoietin-stimulating agents (ESA) and iron supplementation [4]. ESAs mimic human EPO to promote the synthesis of RBCs and bring Hb levels back to normal in anemic individuals. However, ESAs are more likely to develop major side effects like stroke, epilepsy, vascular thrombosis, myocardial infarction, and even death. Also, the development of renal disease, hospitalizations due to cardiovascular diseases, and mortality have not improved with ESA usage. Therefore, iron supplementation is done during the ESA trials to minimize the ESA dosage, but this action increases the risk of allergies, infections, and even iron overload. Additionally, it was found that 10-20% of patients with CKD-related anemia are immune to ESAs [5]. Hence, the advancement of alternative modalities could be beneficial for CKD patients. Hypoxia-inducible factor-prolyl hydroxylase domain inhibitors (HIF-PHIs) have the potential as an alternative to ESAs. These agents inhibit the degradation of the HIF to promote erythropoiesis by stimulating the body’s physiological hypoxic condition [6]. By preventing the prolyl hydroxylase enzymes from functioning, HIF-PHIs stabilize and support the expression of the HIF-1 and HIF-2 genes, which help increase the synthesis of EPO and regulation of iron homeostasis. The latter ability is their advantage over ESAs. Various RCTs have individually discovered that HIF-PHIs substantially benefit iron regulation in treating anemia by decreasing hepcidin, transferrin saturation (TSAT), and ferritin while increasing transferrin and total iron-binding capacity (TIBC). This effect of HIF-PHIs has a marked effect in treating refractory anemia conditions usually associated with CKD. Also, HIF-PHIs, especially roxadustat, have fewer side effects than ESA and are more efficacious in therapy for anemia in CKD [7]. The primary goal of this meta-analysis is to comprehensively evaluate the safety and efficacy of HIF-PHIs compared to ESAs in treating anemia in CKD patients. Notably, the small sample sizes have remained a common restriction for most clinical trials restricted to phase II or III. By systematically synthesizing data from multiple RCTs, this study aims to provide a robust assessment of the relative benefits, risks, and treatment effects of HIF-PHIs and ESAs, overcoming the limitations of individual studies. The clinical implications of this research could be substantial, as it informs evidence-based decision-making in clinical practice guidelines and potentially leads to improved patient outcomes and quality of life. Therefore, to ascertain their effects on the correction of anemia, regulation of iron metabolism, and the incidence of adverse events, we performed a systematic review and meta-analysis of 19 studies to enumerate the beneficial effects and safety concerns of HIF-PHIs over ESAs. Furthermore, this meta-analysis will contribute to the existing body of knowledge on anemia management in CKD patients, identify literature gaps, and help highlight areas where further research is needed.

## Review

Methodology

This meta-analysis follows the Preferred Reporting Items for Systemic Review and Meta-Analysis (PRISMA) guidelines [8].

Data Sources and Search Strategy

An intensive search was conducted on PubMed, Google Scholar, and Embase databases for various clinical studies (updated in May 2023). Using medical subject heading (MeSH) terms and straightforward keyword combinations (such as "anemia," "CKD," "HIF-PHI," "EPO stimulating agents," "safety," and "efficacy"), relevant literature was retrieved. Detailed information on the search strategy is mentioned in Appendix 1. Based on the search, we found 2,555 studies in PubMed. Out of the total studies found, 65 studies were of metanalysis. The databases were searched for published studies in English, including systematic reviews and meta-analyses. The population, intervention, comparison, and outcomes (PICO) approach was utilized. Two reviewers (Nanush Damarlapally and Hamza Irfan) checked the paper's abstract, texts, and titles. The relevant studies were imported into Endnote X9 (Clarivate Analytics, US) to eliminate duplications.

Eligibility Criteria

Inclusion criteria: This meta-analysis included English publications that satisfied specific parameters. We incorporated completed RCTs that focused on individuals diagnosed with CKD, irrespective of their dialysis status, who also exhibited anemia. Eligible patients must have undergone high-intensity focused iron (HIFI) treatment in the experimental group or received ESA, specifically epoetin alpha, as standard care or a placebo in the control group. The studies needed to examine outcomes such as changes in hemoglobin (Hb) levels, Hb response, mean ferritin, mean total iron-binding capacity (TIBC), mean transferrin, and mean iron levels. Additionally, we considered drug safety events, including major adverse cardiovascular effects (MACE), hypertension, nausea, vomiting, diarrhea, hyperkalemia, serious adverse events, progression of CKD, and muscle spasms. Our stringent inclusion criteria ensured the reliability and robustness of our meta-analysis results.

Exclusion criteria: To maintain the integrity and rigor of this meta-analysis, a set of stringent exclusion criteria was diligently applied. Firstly, nonclinical studies were unequivocally excluded from consideration. Furthermore, studies lacking precise and well-defined criteria for diagnosing CKD accompanied by anemia were omitted from the analysis. To maintain the highest standards of evidence, any form of case-based literature, including case reports, case sheets, case series studies, editorial pieces, and review articles, was excluded from the review process. Duplicate publications previously assessed or published were also carefully screened out to avoid redundancy. Equivocal study results that did not provide transparent and interpretable findings were not considered. Similarly, studies lacking comparable groups or controls were excluded to ensure a robust comparative analysis. Additionally, studies with incomplete or unavailable full texts were not incorporated into the analysis. Lastly, studies with a sample size of less than 20 were excluded to uphold statistical significance and minimize potential biases. These meticulous exclusion criteria were implemented to guarantee the credibility and validity of the findings derived from this meta-analysis.

Data Extraction

Two researchers, Nanush Damarlapally and Hamza Irfan, independently assessed the selected studies to determine whether a particular article should be included based on inclusion and exclusion criteria. A dialogue examined and resolved uncertain data. The following essential variables were retrieved from each study: first author's name, publication year, characteristics of participants, patients with CKD, type of erythropoietic agent used, and type of HIFI or ESA. The respective information was included in three different tables.

Clinical outcomes: Primary outcomes included changes in Hb and Hb response. Secondary efficacy outcomes comprised changes in mean hepcidin, mean iron, mean IV monthly iron used, mean ferritin, TIBC, and mean transferrin. Secondary safety outcomes encompassed adverse events, drug-related adverse events, adverse events leading to withdrawal, MACE, hyperkalemia, stroke, vomiting, back pain, diarrhea, pneumonia, hypotension, cancer-related deaths, nasopharyngitis, retinal changes, progression of CKD, and muscle spasms.

Study Quality Assessment

The quality assessment of published RCTs was conducted using the revised Cochrane Collaboration's risk of bias [9]. Reviewers assessed the risk for bias in the trial according to standard criteria (randomization of subjects, allocation concealment, blinding of patient, investigators and outcome assessors, completeness to follow up, and use of intention to treat analysis, measurement of the values, and any fundings involved). It resolved any discrepancies in data extraction by a discussion with an arbitrator.

Data Analysis

The statistical analysis was conducted using RevMan (version 5.4; Copenhagen: The Nordic Cochrane Centre, The Cochrane Collaboration, 2014) software. Relative risks (RRs) and their respective 95% CI were retrieved for dichotomous outcomes. Continuous outcomes were given with mean values and SDs. Forest plots were generated for the visual presentation of the data, and funnel plots for all outcomes were used to assess publication bias. All p-values less than 0.05 were considered statistically significant. HIF-PHIs were sub-grouped for primary outcomes such as mean Hb and Hb response changes. Heterogeneity between trials was quantified and presented as a percentage using I2 statistics. An I2 value between 0% and 40% suggested low or non-important heterogeneity, an I2 value between 30% and 60% indicated potential moderate heterogeneity, an I2 value between 50% and 90% suggested substantial heterogeneity, and an I2 value between 75% and 100% denoted considerable heterogeneity [10]. A sensitivity analysis was conducted on data from studies with heterogeneity greater than 75% to determine the influence of each study on the pooled estimate. Baseline variables, such as age and mean Hb, were used as covariates for comparison with our primary outcomes through meta-regression, and the results were tabulated. Seventeen and fourteen studies reported changes in mean Hb and Hb response, respectively.

Results

Study Selection

**Figure 1:**
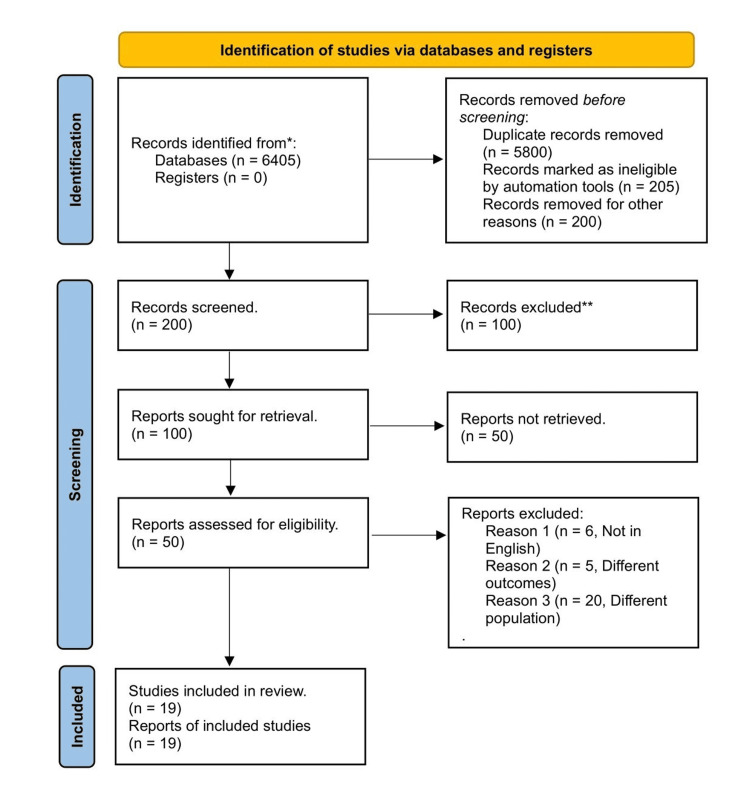
PRISMA flow chart showing search strategy and study selection process. PRISMA: Preferred Reporting Items for Systemic Review and Meta-Analysis.

A total of 16,255 articles were identified from the preliminary literature search. After eliminating duplicated articles and based on title and abstract, a total of 19 RCT studies were included in this meta-analysis [11-29]. The PRISMA diagram illustrates a comprehensive search strategy, as shown in Figure 1. It includes the collection of studies from 2016 through 2022.

Baseline Characteristics

In the 19 studies, a total of 22,151 CKD patients were randomly assigned, with 11,234 receiving HIF-PHIs and 10,917 receiving ESA. The mean ages of patients in both groups were between 56.21 ± 11.2 and 60.01 ± 12.5 years, respectively. Most of the population was male, with 53.35% (n= 5994) in HIF-PHIs and 64.88% (n = 5809) in the ESA groups. The baseline values for the primary and secondary outcomes, racial and regional differences, cardiovascular parameters, renal parameters, dialysis-related parameters, and systemic illnesses like diabetes and glomerulonephritis are all mentioned in Tables 1-3. The follow‐up duration of the study subjects ranged from 24 to 104 weeks.

**Table 1:** Baseline characteristics of the study subjects, including the number of patients with or without dialysis, type of study, study design, and blood-related parameters. Hb: haemoglobin; HIF-PHI: Hypoxia-inducible factor-prolyl hydroxylase domain inhibitors; ESA: Erythropoietin-stimulating agents; TSAT: Transferrin saturation.

Study	Study Design	Number of Patients		Total Patients	Age (years) (Mean ± SD)		Hb (g/dl) (Mean ± SD)		Hepcidin (ng/ml) (Mean ± SD)		TSAT % (Mean ± SD)		Ferritin (ng/ml) (Mean ± SD)	
	Type	Dialysis	Non-Dialysis		HIF-PHI	ESA	HIF-PHI	ESA	HIF-PHI	ESA	HIF-PHI	ESA	HIF-PHI	ESA
Singh (B) 2021 [11]	RCT	N/A	3872	3872	67 ± 3.3	67 ± 3.1	9.9 ± 0.9	9.8 ± 0.9	105.6 ± 19.3	105.3 ± 20.1	30 ± 2.4	29 ± 2.4	267 ± 54.0	275 ± 51.4
Singh (A) 2021 [12]	RCT	2964	N/A	2964	58 ± 3.5	59 ± 3.8	10.3 ± 0.9	10.3 ± 0.9	172.7 ± 27.1	179.6 ± 26.5	33 ± 2.7	32 ± 2.9	589 ± 117.0	604 ± 112.4
Yamamoto A 2021 [13]	RCT	N/A	N/A	164	69 ± 10.3	72.4 ± 10.3	11.3 ± 0.6	11.2 ± 0.6	N/A	N/A	34 ± 13.6	34.2 ± 9.7	118.2 ± 106.2	124.5 ± 79
Gang 2022 [14]	RCT	392	N/A	392	51.0 ± 13.9	50.9 ± 13.4	9.6 ± 0.9	9.5 ± 1.3	15.6 ± 10.3	16.6 ± 94.9	37.6 ± 18.5	35.8 ± 16.4	1,209.7 ± 1,133.8	1,188.5 ± 1,170.5
Singh C 2022 [15]	RCT	312	N/A	312	52 ± 3.3	56 ± 3.7	9.5 ± 1	9.5 ± 1	118.4 ± 22.5	124.8 ± 30.9	28 ± 2.2	30 ± 2.2	365 ± 54.9	373 ± 76.1
Barrat 2021 [16]	RCT	N/A	616	616	66.8 ± 13.6	65.7 ± 14.4	9.5 ± 0.75	9.5 ± 0.6	N/A	N/A	24 ± 10.1	23.2 ± 10.6	233.7 ± 231.21	225.0 ± 207.7
Eckardt 2021 [17]	RCT	3923	N/A	3923	57.7 ± 13.9	58.1 ± 13.8	10.5 ± 0.9	10.1 ± 0.8	187.3 ± 137.5	184.3 ± 133.7	37.5 ± 13.2	37.3 ± 12.6	811.9 ± 544.7	810.7 ± 526.9
Coyne 2022 [18]	RCT	407	N/A	407	60 ± 3.5	56 ± 3.5	10.5 ± 0.1	10.7 ± 0.2	159 ± 26.8	176 ± 26.8	32 ± 2.7	35 ± 2.9	589 ± 110.9	553 ± 102.5
Akiwaza A 2020 [19]	RCT	N/A	N/A	303	64.6 ± 11.7	64.9 ± 10.1	11.0 ± 0.56	11.01 ± 0.6	N/A	N/A	28.2 ± 11.7	29.0 ± 10.1	102.3 ± 83.4	96.2 ± 75.1
Chen 2019 [20]	RCT	N/A	N/A	305	47.6 ± 11.7	51 ± 11.8	10.4 ± 0.7	10.5 ± 0.7	180.7 ± 136.8	148.3 ± 104.2	33.8 ± 16.6	30 ± 13.8	498.5 ± 487.4	420.1 ± 406.8
Fishbane 2022 [21]	RCT	2106	N/A	2106	53.5 ± 15.3	54.5 ± 15	10.2 ± 0.2	10.3 ± 0.3	275.6	269.6	36	34.9	542.9	555.7
Akizawa B 2020 [22]	RCT	271	N/A	271	64 ± 10	64 ± 11	10.9 ± 0.8	10.8 ± 0.7	54.1 ± 58.3	60.1 ± 63.1	27 ± 10.1	26.3 ± 8.9	86.5 ± 94.1	96.6 ± 120.2
Yamamoto B 2021 [23]	RCT	N/A	162	162	72.1 ± 9.3	71.2 ± 10.1	9.8 ± 0.6	10 ± 0.6	N/A	N/A	28.5 ± 8.5	29.9 ± 9.7	118.9 ± 97.4	137.9 ± 113.9
Provenzano A 2016 [24]	RCT	90	N/A	90	56.9 ± 12.1	57 ± 11.6	11.2 ± 0.7	11.2 ± 1	327.1 ± 178.8	298.7 ± 123.1	29.2 ± 10	28.6 ± 14.4	827.7 ± 474.3	1106.6 ± 642.1
Nangaku 2021 [25]	RCT	323	N/A	323	66 ± 11.3	64.9 ± 11.7	10.6 ± 10.4	10.6 ± 10.5	N/A	N/A	28.6 ± 10.6	26.9 ± 9.4	144.5 ± 139.6	140 ± 95.3
Agrawal 2022 [26]	RCT	N/A	588	588	53.3 ± 13.9	52.1 ± 13.6	8.99 ± 0.78	8.99 ± 0.74	59.2 ± 50.7	59.4 ± 54.0	27.2 ± 12.7	27.1 ± 13.6	421.1 ± 526.8	408.5 ± 624.1
Chertow 2021 [27]	RCT	N/A	1725	3476	67.3 ± 13.1	66.5 ± 13.5	N/A	N/A	N/A	N/A	N/A	N/A	N/A	N/A
Csiky 2021 [28]	RCT	834	N/A	834	61 ± 13.8	61.8 ± 13.4	10.7 ± 0.6	10.7 ± 0.6	N/A	N/A	N/A	N/A	N/A	N/A
Provenzano B 2021 [29]	RCT	1043	N/A	1043	53.8 ± 14.7	54.3 ± 14.6	8.4 ± 1	8.5 ± 1	N/A	N/A	27 ± 9.3	27.6 ± 8.9	441.4 ± 337	437.4 ± 311.4

**Table 2:** Baseline characteristics of the study subjects, including duration of the study, type of drugs used, race, and other blood-related parameters measured. Wks: Weeks; HIF-PHI: Hypoxia-inducible factor-prolyl hydroxylase domain inhibitors; ESA: Erythropoietin-stimulating agents; Dp: Daprodustat; DA: Darbepoetin alfa; TIBC: Total iron-binding capacity; Md: Molidustat; De: Desidustat; EA: Erythropoietin alfa; Rd: Roxadustat; Vd: Vadadustat; Y: Yes; N: No; N/A: Not available; H/o: History of; No: Number; %: Percentage.

Study	Time (weeks)	Drug used		H/o Use of ESA	African American no. (%)		Weight (Kg) (Mean ± SD)		Iron (Μmol/L) (Mean ± SD)		Iron IV no. (%)		Iron Dose (mg) (Mean ± SD)		TIBC (μmol/ L) (Mean ± SD)	
		HIF-PHI	ESA		HIF-PHI	ESA	HIF-PHI	ESA	HIF-PHI	ESA	HIF-PHI	ESA	HIF-PHI	ESA	HIF-PHI	ESA
Singh (B) 2021 [11]	52	Dp	DA	Y	183 (9.4)	185 (9.6)	N/A	N/A	13 ± 1.1	13 ± 1.11	226 (1.7)	228 (11.8)	N/A	N/A	45 ± 1.8	44 ± 1.6
Singh (A) 2021 [12]	52	Dp	DA	Y	228 (15.3)	233 (15.8)	N/A	N/A	13 ± 1.11	13 ± 1.11	956 (64.3)	943 (63.8)	139.2 ± 171.1	137.4 ± 174.7	39 ± 1.67	39 ± 1.67
Yamamoto A 2021 [13]	52	Md	DA	Y	N/A	N/A	60.6 ± 11.3	60.1 ± 11.0	N/A	N/A	1.0 (1.09)	2.2 (3.1)	N/A	N/A	N/A	N/A
Gang 2022 [14]	24	De	EA	Y	N/A	N/A	58.9 ± 13.3	60.2 ± 13.3	N/A	N/A	N/A	N/A	N/A	N/A	N/A	N/A
Singh C 2022 [15]	52	Dp	DA	N/A	16 (10)	13 (8)	75 ± 4.5	74 ± 4.5	N/A	N/A	105 (67)	109 (70)	159.3 ± 207.1	180.1 ± 209.9	N/A	N/A
Barrat 2021 [16]	104	Rd	DA	N/A	8 (2.5)	2 (0.7)	76.9 ± 16.3	78.3 ± 17.6	11.2 ± 4.6	10.7 ± 4.3	47 (14.6)	39 (13.3)	N/A	N/A	N/A	N/A
Eckardt 2021 [17]	52	Vd	DA	N/A	398.3 (24)	404.8 (24.5)	N/A	N/A	N/A	N/A	835.3 (21.8)	781.5 (49.1)	N/A	N/A	N/A	N/A
Coyne 2022 [18]	52	Dp	EA	N/A	49 (18)	32 (23)	N/A	N/A	42.8 ± 3.5	42.87 ± 3.7	N/A	N/A	N/A	N/A	212 ± 8.3	212 ± 8.3
Akiwaza A 2020 [19]	24	Rd	DA	N/A	N/A	N/A	57.8 ± 11.9	58.7 ± 12.9	12.1 ± 5.1	12.6 ± 4.5	N/A	N/A	N/A	N/A	N/A	N/A
Chen 2019 [20]	27	Rd	EA	Y	N/A	N/A	62.8 ± 11.8	61.5 ± 9.9	N/A	N/A	N/A	N/A	N/A	N/A	47.4 ± 11.4	48.3 ± 9
Fishbane 2022 [21]	52	Rd	DA	N/A	148 (14.1)	158 (15)	75.1 ± 21.2	75.1 ± 19.7	44.1	43.07	885 (84.2)	920 (87.2)	58.71	91.37	10.05	10.08
Akizawa B 2020 [22]	52	Dp	DA	Y	N/A	N/A	N/A	N/A	39.9 ± 14.6	38.1 ± 13.5	32 (11.8)	43 (15.9)	14	17	12.3 ± 1.8	12.0 ± 1.8
Yamamoto B 2021 [23]	52	Md	DA	Y	N/A	N/A	61.1 ± 10	60.4 ± 10.4	NA	N/A	4 (4.9)	4 (5)	2.8 ± 4.1	11.2 ± 20.4	N/A	N/A
Provenzano A 2016 [24]	19	Rd	EA	N/A	29 (43)	12 (52)	86.6 ± 22.5	84.3 ± 23.4	38.99 ± 12.1	37.1 ± 18.7	N/A	N/A	N/A	N/A	9.6 ± 1.6	9.7 ± 1.3
Nangaku 2021 [25]	52	Vd	DA	Y	N/A	N/A	58.1 ± 11.9	58.8 ± 13.8	N/A	39.6 ± 22.7	N/A	N/A	N/A	N/A	N/A	N/A
Agrawal 2022 [26]	24	De	DA	Y	N/A	N/A	60.9 ± 10.5	62.6 ± 13.3	38.2 ± 17.4	N/A	N/A	N/A	N/A	N/A	N/A	N/A
Chertow 2021 [27]	52	Vd	DA	Y	93 (10)	131 (15)	N/A	N/A	N/A	N/A	N/A	N/A	N/A	N/A	N/A	N/A
Csiky 2021 [28]	52-104	Rd	DA	Y	6 (1.4)	6 (1.4)	N/A	N/A	N/A	N/A	N/A	N/A	N/A	N/A	N/A	N/A
Provenzano B 2021 [29]	52	Rd	EA	N	44 (8)	50 (9)	76 ± 18	76.7 ± 19	N/A	N/A	N/A	N/A	N/A	N/A	N/A	N/A

**Table 3:** Baseline characteristics showing details of the study subjects, including blood pressure, cholesterol levels, and the number of subjects with hypertension, diabetes, cardiovascular diseases, and those on hemodialysis. HIF-PHI: Hypoxia-inducible factor-prolyl hydroxylase domain inhibitors; ESA: Erythropoietin-stimulating agents; No: Number; SBP: Systolic blood pressure; CVD: Cardiovascular disease; DSP: Diastolic blood pressure; LDL: Low-density lipoproteins; HDL: High-density lipoproteins; HTN: Hypertension; DM: Diabetes mellitus.

Study	SBP (Mean ± SD)		DBP (Mean ± SD)		LDL mg/dl (Mean ± SD)		HDL mg/dl (Mean ± SD)		HTN no. (%)		CVD no. (%)		DM no. (%)		Haemo-dialysis no. (%)	
	HIF-PHI	ESA	HIF-PHI	ESA	HIF-PHI	ESA	HIF-PHI	ESA	HIF-PHI	ESA	HIF-PHI	ESA	HIF-PHI	ESA	HIF-PHI	ESA
Singh (B) 2021 [11]	N/A	N/A	N/A	N/A	84.2 ± 8.65	84.2 ± 8.35	46.3 ± 3.58	46.3 ± 3.58	N/A	1829 (94.5)	716 (37)	716 (37)	1076 (55.5)	1118 (57.8)	N/A	N/A
Singh (A) 2021 [12]	N/A	N/A	N/A	N/A	81.9 ± 7.8	81.1 ± 7.95	40.5 ± 3.22	40.5 ± 3.7	152.5 (10.7)	1373 (93)	666 (44.8)	665 (45)	615 (41.4)	67 (41.8)	1316 (88.5)	1308 (88.6)
Yamamoto A 2021 [13]	132.3 ± 16.1	132.9±16.5	71.5 ± 13.3	70.4 ± 10.6	N/A	N/A	N/A	N/A	N/A	N/A	N/A	N/A	N/A	N/A	N/A	N/A
Gang 2022 [14]	143.0 ± 16.7	143.1±14.9	82.0 ± 10.1	82.2 ± 9.6	77.4 ± 31.7	80.3 ± 32.6	40.9 ± 14.1	39.0 ± 12.4	137.0 (37.5)	178 (90.8)	10 (5.1)	7 (3.5)	71 (36.2)	70 (35.7)	N/A	N/A
Singh C 2022 [15]	139 ± 4.45	140 ± 6.2	79 ± 2.5	76 ± 2.7	91.9 ± 7.5	90 ± 6.8	46.3 ± 3.9	44.4 ± 2.8	164.1 (9.6)	N/A	47 (30)	45 (29)	70 (45)	70 (45)	126 (80)	126 (81)
Barrat 2021 [16]	137.1±15.1	137.5±14.8	74.8 ± 9.9	75.2 ± 10.4	N/A	N/A	N/A	N/A	N/A	N/A	N/A	N/A	N/A	N/A	N/A	N/A
Eckardt 2021 [17]	142.8 ± 21.3	143.7 ± 20.6	76.3 ± 13.2	76.1 ± 13.1	N/A	N/A	N/A	N/A	N/A	N/A	794.1 (47.9)	849.8 (51.3)	890.9 (54.9)	911.7 (55.9)	1513.8 (92.5)	1492 (91.8)
Coyne 2022 [18]	135 ± 5.74	133 ± 6.6	74 ± 3.5	76 ± 3.3	N/A	N/A	N/A	N/A	N/A	N/A	110 (41)	54 (39)	105 (39)	53 (39)	N/A	N/A
Akiwaza A 2020 [19]	N/A	N/A	N/A	N/A	N/A	N/A	N/A	N/A	N/A	N/A	N/A	N/A	54 (36)	54 (35.8)	N/A	N/A
Chen 2019 [20]	148.1±16.1	148.4 ± 16.5	85.3 ± 9.8	84.2 ±10.7	95.1 ± 34.8	90.1 ± 29.4	43.3 ± 12	44.5 ± 15.1	168.2 (42.9)	N/A	N/A	N/A	30 (14.7)	17 (17)	182 (89.2)	89 (89)
Fishbane 2022 [21]	140.8±16.9	140.6±17.2	78.2 ± 10.1	77.9 ± 10.3	82 ± 9.12	82 ± 10	N/A	N/A	N/A	1009 (95.6)	N/A	N/A	421 (40.1)	423 (40.1)	111 (10.6)	117 (11.1)
)Akizawa B 2020 [22]	139 ± 23	137 ± 23	78 ± 14	78 ± 13	N/A	N/A	N/A	N/A	N/A	125 (93)	N/A	N/A	56 (41)	52 (39)	86 (63)	88 (65)
Yamamoto B 2021 [23]	138.2±17	133.3±15.8	71.6 ± 13.2	69.1 ± 10.2	99.6±29.2	100.5 ± 31.2	50.8±16.3	51.7 ± 16.6	N/A	N/A	N/A	N/A	N/A	N/A	N/A	N/A
Provenzano A 2016 [24]	N/A	N/A	N/A	N/A	N/A	N/A	N/A	N/A	N/A	22 (100)	N/A	N/A	39 (59)	14 (64)	N/A	N/A
Nangaku 2021 [25]	N/A	N/A	81.1 ± 7.4	74.2 ± 10.9	N/A	N/A	N/A	N/A	N/A	147 (91.3)	N/A	N/A	35 (21.6)	49 (30.4)	N/A	N/A
Agrawal 2022 [26]	132.8±13.6	133.1±13.2	73.4 ± 11.4	74.3 ± 11.2	92.6±41.7	93.2 ± 41	40.6±13.9	40.7 ± 12.2	158.7 (50.4)	240 (81.6)	19 (6.4)	15 (5.1)	140 (47.6)	145 (49.3)	N/A	N/A
Chertow 2021 [27]	137.1 ± 18	136.4 ± 17.5	75.2 ± 11	N/A	N/A	N/A	N/A	N/A	N/A	N/A	375 (43.5)	402 (46.6)	517 (60)	518 (60)	N/A	N/A
Csiky 2021 [28]	135.2 ± 17.6	136.9 ± 18.9	N/A	N/A	N/A	N/A	N/A	N/A	N/A	N/A	N/A	N/A	104 (25)	133 (31.7)	N/A	N/A
Provenzano B 2021 [29]	N/A	N/A	N/A	N/A	109.1 ± 38.8	109.2 ± 35.9	N/A	N/A	184.6 (45)	504 (96)	N/A	N/A	205 (39)	204 (39)	N/A	N/A

Quality Assessment and Publication Bias

**Figure 2:**
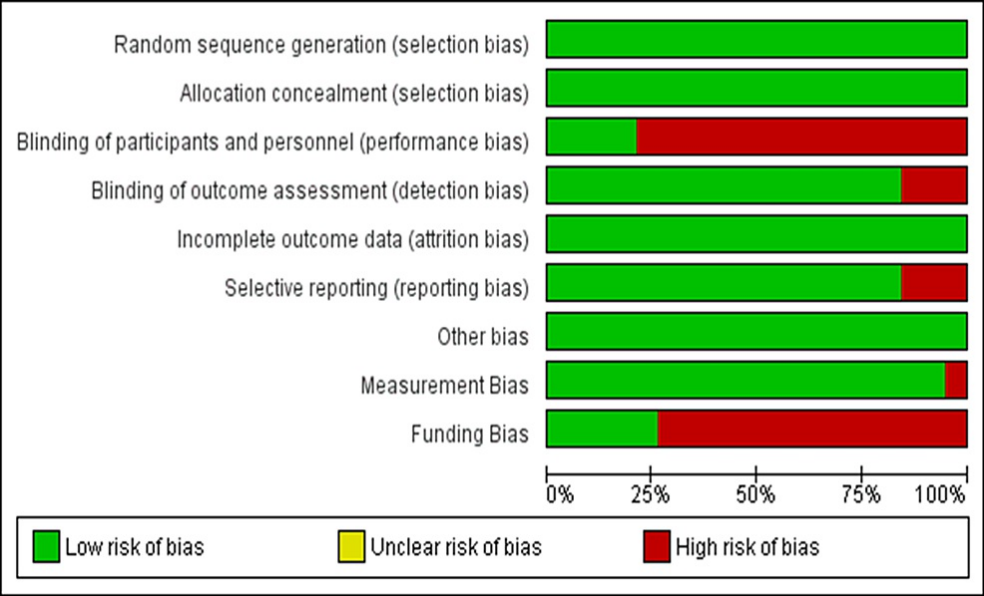
Cochrane risk of bias tool for assessing publication bias in randomized controlled trials.

**Figure 3:**
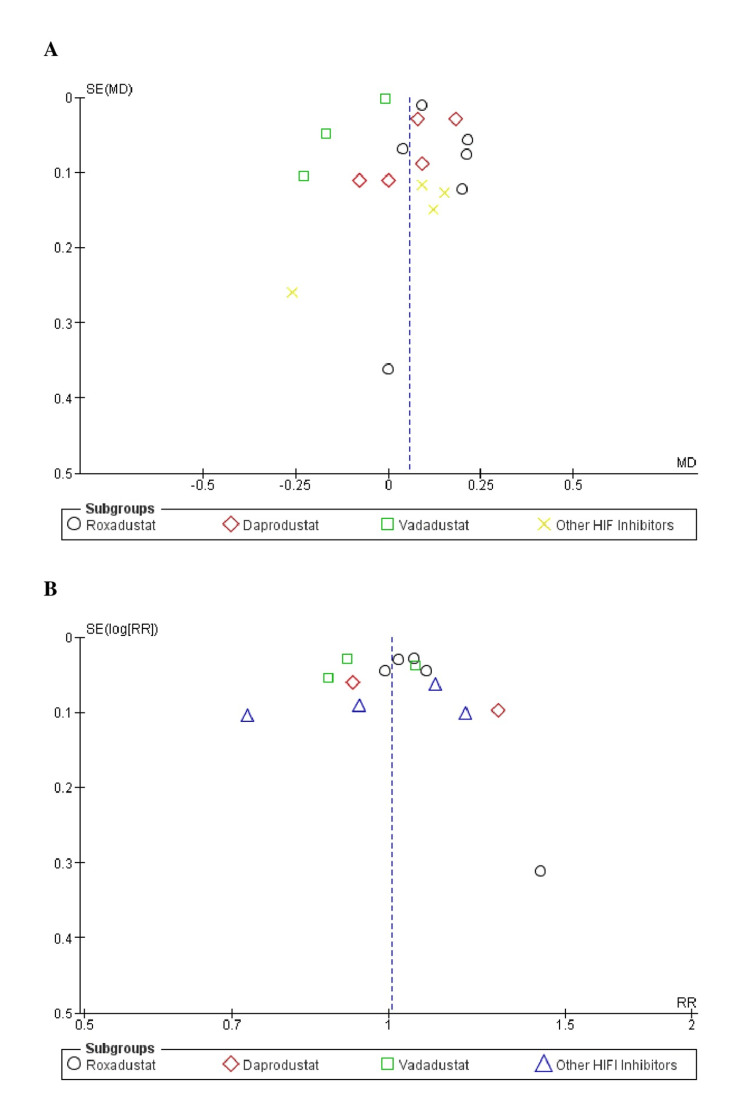
Funnel plot of (a) change in mean Hb, and (b) Hb response. The above funnel plot clearly shows there was no publication bias in the study. MD: Mean difference; RR: Relative risk; SE: Standard error.

Using the Cochrane risk bias tool to assess the quality of studies, it was determined that the RCT studies had a minimal risk of bias (Figure 2). The funnel plots of primary outcomes demonstrated that publication bias did not affect the quantitative results (Figure 3).

Primary Outcomes

**Figure 4:**
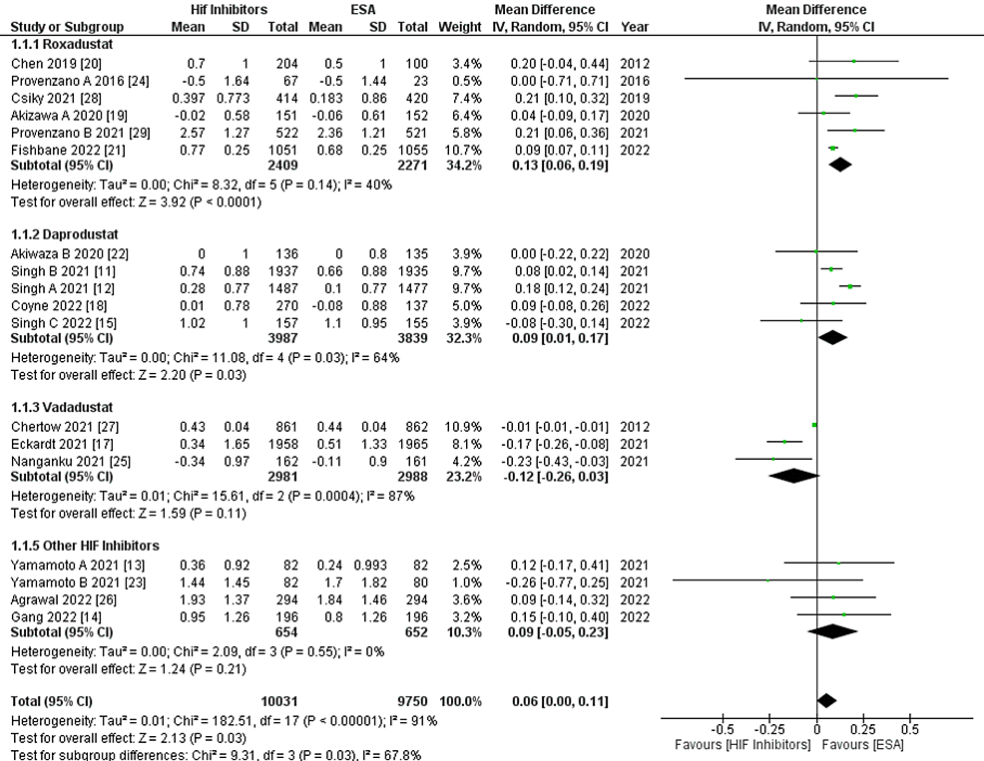
Forest plot showing subgroup analysis of change in mean Hb (g/dL) by HIF-PHI vs. ESA drugs. The forest plot above shows a significant increase in the change in mean Hb for roxadustat (MD: 0.13, 95% CI: 0.06, 0.19; p: 0.0001, I2: 40%) and daprodustat (MD: 0.09, 95% CI: 0.01, 0.17; p: 0.03, I2: 64%), and a non-significant increase for molidustat and desidustat (MD: 0.09, 95% CI: -0.05, 0.23; p: 0.21, I2: 0%), respectively. MD: Mean difference; HIF-PHI: Hypoxia-inducible factor-prolyl hydroxylase domain inhibitors; ESA: Erythropoietin-stimulating agents; Hb: Hemoglobin. Source: References [11-15, 17-29].

Change in mean Hb (g/dl): In a pooled analysis of 18 studies, it was found that various HIF-PHIs showed a significant overall increase in the change in mean Hb compared to ESAs (MD: 0.06, 95% CI: 0.00, 0.11; p: 0.03, I²: 91%), as shown in Figure 4. Given the high heterogeneity, we conducted subgrouping, meta-regression, and leave-one-out-study analysis.

When subgrouping the outcomes by different HIF-PHI, data from six studies showed that roxadustat led to a significantly greater increase in the change in mean Hb (MD: 0.13, 95% CI: 0.06, 0.19; p: 0.0001, I2: 40%), as given in Figure 4. This was followed by daprodustat, used in five studies (MD: 0.09, 95% CI: 0.01, 0.17; p: 0.03, I2: 64%), shown in Figure 4. Meanwhile, four studies using other HIF-PHI (including molidustat and desidustat) showed a non-significant increase in the change in mean Hb (MD: 0.09, 95% CI: -0.05, 0.23; p: 0.21, I2: 0%), shown in Figure 4. Interestingly, three studies using vadadustat showed a non-significant decrease in the change in mean Hb (MD: -0.12 95% CI: -0.26, 0.03; p: 0.11, I2: 87%), as shown in Figure 4. The subgroup differences in outcomes between different HIF-PHI from 17 studies were found to be significant (Chi²: 9.31, df: 3, P: 0.03, I2: 67.8%), as shown in Figure 4.

Due to high heterogeneity, we employed age and mean Hb as covariates and performed univariate meta-regression for change in Hb (Table 4). This analysis revealed that age had a lesser non-significant association (coefficient value: -0.007, p: 0.204) with the change in mean Hb. In contrast, mean Hb as a covariate showed no association and was statistically non-significant (coefficient value: 0.00, p: 0.878).

**Table 4:** Univariate meta-regression analysis showing the effect of age and mean Hb on primary outcomes: change in mean Hb (g/dL) and Hb response (n%). The analysis indicates that age as a covariate has a minor, non-significant association with changes in hemoglobin (coefficient value: -0.007, p: 0.204) and hemoglobin response (coefficient value: -0.008, p: 0.062), while mean hemoglobin as a covariate shows no association with changes in hemoglobin (coefficient value: 0.00, p: 0.878) and a slight, non-significant association with changes in hemoglobin response (coefficient value: 0.012, p: 0.756). Hb: Hamoglobin. N: number.

Outcomes	Covariate	Co-efficient	P-value
Change in Hb (g/dl)	Age	-0.007	0.204
	Mean Hb	0.000	0.878
Hb Response N (%)	Age	-0.008	0.062
	Mean Hb	-0.012	0.756

Finally, we performed a sensitivity analysis by excluding the study by Chertow GM et al. [27], which reduced the overall heterogeneity (MD: 0.07; 95% CI: 0.01-0.12; P: 0.02; I2: 75%).

**Figure 5:**
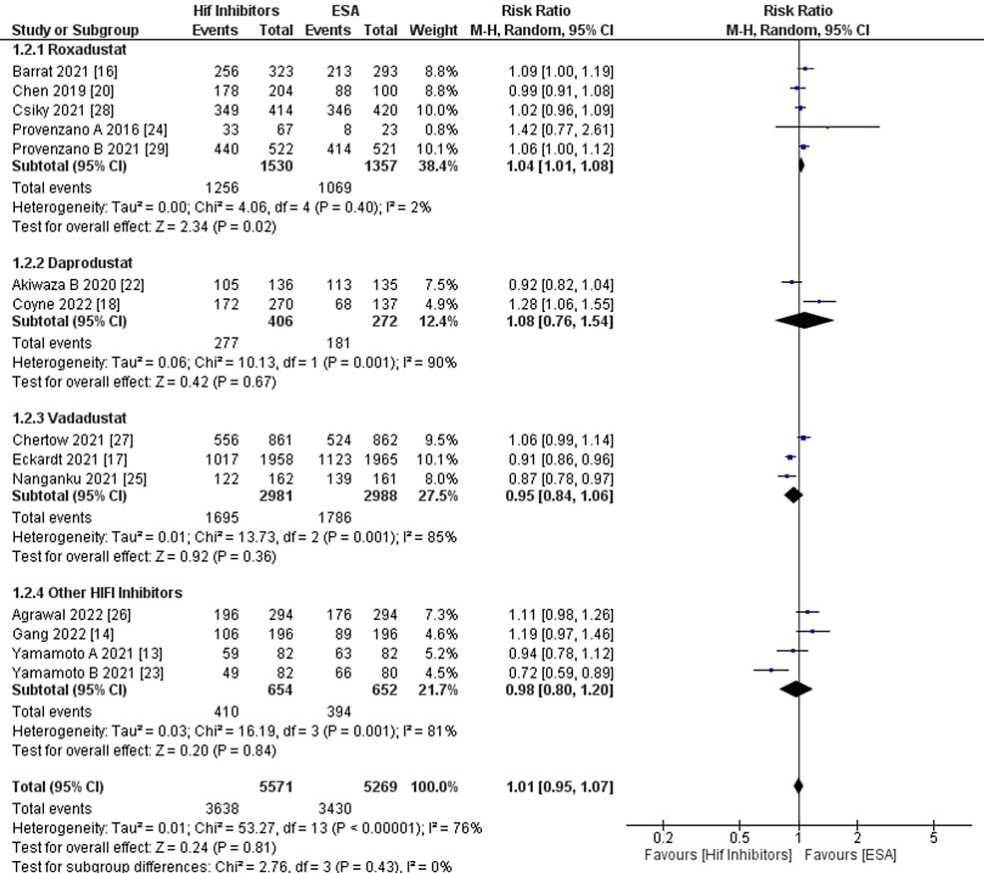
Forest plot showing subgroup analysis of Hb response by HIF-PHI vs. ESA drugs. The forest plot above shows that various HIF-PHIs demonstrated an overall non-significant difference in Hb response (RR: 1.01, 95% CI: 0.95, 1.07; p: 0.81, I²: 76%) compared to ESAs. RR: Relative risk ratio; HIFI: Hypoxia-inducible factor-prolyl hydroxylase domain inhibitors; ESA: Erythropoietin-stimulating Agents; Hb: Haemoglobin. Source: References [13, 14, 16-18, 20, 22-29].

Change in Hb response: A pooled analysis of 12 studies revealed no significant difference in Hb response when various HIF-PHIs were compared to ESAs, showing an overall non-significant result (RR: 1.01; 95% CI: 0.95-1.07; p: 0.81; I²: 76%; Figure 5). Due to the high heterogeneity, we conducted subgroup analyses, meta-regression, and sensitivity analyses. Upon subdividing the outcomes by specific HIF-PHIs, five studies involving roxadustat indicated a slightly higher, yet significant, Hb response (RR: 1.04; 95% CI: 1.01-1.08; p: 0.02; I2: 2%; Figure 5). In contrast, two studies on daprodustat demonstrated a non-significant and slight increase in Hb response (RR: 1.08; 95% CI: 0.76-1.54; p: 0.67; I2: 90%; Figure 5), while four studies examining other HIF-PHIs (including molidustat and desidustat) revealed a non-significant decrease in Hb response (RR: 0.98; 95% CI: 0.80-1.20; p: 0.84; I2: 81%; Figure 5). Additionally, three combined studies on vadadustat showed a non-significant decrease in Hb response (RR: 0.95; 95% CI: 0.84-1.06; p: 0.36; I2: 85%; Figure 5). Overall subgroup differences in outcomes between different HIF-PHIs among 12 studies were found to be non-significant (Chi²: 2.76, df: 3, P: 0.43, I2: 0%, Figure 5). As per Table 4, we used age and mean Hb as covariates and performed univariate meta-regression for Hb response and found that age has a lesser non-significant association (coefficient value: -0.008, P: 0.062) with Hb Response while mean Hb as a covariate showed slight non-significant association (coefficient value: 0.012 P: 0.756). Sensitivity analysis performed by excluding Kai et al. study [14] decreased the overall heterogeneity (RR: 1.02, 95% CI: 0.96, 1.08 p: 0.52, I2: 70%) but showed non-significant (slightly) increased risk.

In conclusion, the data suggests that while HIF-PHI might produce a slight increase in mean Hb concentration compared to ESAs, there is no significant difference in achieving a desired Hb response between these two groups. However, the significant heterogeneity (I2 > 75%) in many comparisons suggests that the studies included may be quite diverse in their characteristics or qualities.

Secondary Efficacy Outcomes

This study analyzed the efficacy outcomes of various HIF-PHI in comparison with ESA. Detailed information on RR, 95% CI, p-values, and I2 are noted in Table 5. Subgroup analysis was conducted for all efficacy outcomes such as change in mean ferritin, change in mean hepcidin, change in mean TIBC, change in mean TSAT%, change in mean monthly IV iron, and change in mean iron.

**Table 5:** Secondary efficacy outcomes. The table above demonstrates significant reductions in mean iron, monthly IV iron use, hepcidin, and ferritin, along with a non-significant decrease in TSAT and a significant rise in TIBC, observed with the overall use of HIF-PHI drugs. Both roxadustat and daprodustat contributed to these trends, showing marked decreases in monthly IV iron use and hepcidin, and notable increases in TIBC. CI: Confidence interval; I2: Heterogeneity; TIBC: Total iron-binding capacity; TSAT: Transferrin saturation.

Outcome	Effect Size (Mean difference)	95%CI	P-value	I2
Change in Mean Hepcidin (ng/ml)	-21.04	-28.92, -13.17	<0.00001	96%
Roxadustat	-19.95	-39.39, -0.51	0.04	86%
Daprodustat	-25.02	-34.64, -15.39	<0.00001	98%
Vadadustat	-22.16	-30.19, -14.13	<0.00001	NA
Change in Mean Iron (micromol/L)	-1.54	-3.01, -0.06	0.04	100%
Roxadustat	-1.89	-9.62, 5.84	0.63	100%
Daprodustat	0.78	-0.78, 2.34	0.33	100%
Mean IV Monthly Iron Use (mg)	-18.6	-32.02, 5.19	0.007	90%
Roxadustat	-33.39	-39.13, -27.65	<0.00001	0%
Daprodustat	-9.1	-9.34, -8.86	<0.00001	0%
Change in Mean TSAT (%)	-0.31	-1.79,1.18	0.69	99%
Roxadustat	0.55	-0.23, 1.33	0.17	0%
Daprodustat	-1.27	-3.83, 1.29	0.33	100%
Vadadustat	-2.7	-3.53, -1.87	0.33	100%
Change in Mean Ferritin (ng/ml)	-16.45	-27.17, -5.73	0.003	87%
Roxadustat	-17.62	-44.82, 9.58	0.20	86%
Daprodustat	-15.13	-27.84, -2.42	0.02	93%
Vadadustat	-38.83	-72.83, -4.83	0.03	NA
Change in Mean TIBC (micromol/L)	4.98	3.70, 6.26	<0.00001	100%
Roxadustat	5.5	2.80, 8.19	<0.00001	98%
Daprodustat	4.41	3.29, 5.54	<0.00001	99%

Change in mean ferritin (ng/ml): A significant reduction in the change in mean ferritin was observed across nine studies using HIF-PHI, specifically daprodustat and vadadustat subgroups. However, the roxadustat subgroup demonstrated a non-significant decrease compared to ESAs. A sensitivity analysis performed did not affect the outcome. Change in mean hepcidin (ng/ml): The change in mean hepcidin significantly decreased with the overall use of HIF-PHI in 12 studies. This reduction was consistent across all HIF-PHI subgroups, including daprodustat, vadadustat, roxadustat, and others (molidustat and desidustat). Variations among studies could not be reduced despite conducting a sensitivity analysis analysis. Change in mean TIBC (micromol/L): The study found an overall significant increase in the change in mean TIBC in nine studies. Subgroups like Roxadustat and Daprodustat also showed a statistically significant increase in the change in mean TIBC. However, the heterogeneity among study groups could not be decreased through sensitivity analysis. Change in mean TSAT (%): The change in mean TSAT was not significantly decreased overall in 10 studies and the daprodustat subgroup but significantly decreased in the vadadustat subgroup. Roxadustat studies displayed a non-significant increase in mean TSAT. Sensitivity analysis did not reduce variations due to high heterogeneity. Mean monthly IV iron (mg): Mean monthly IV iron use significantly decreased overall in six studies, specifically in the roxadustat and daprodustat subgroups. The exclusion of the Ajay A et al. [12] study via sensitivity analysis led to a decrease in heterogeneity. Change in mean iron (micromol/L): A significant decrease in the change in mean iron was noted in 10 overall studies and the roxadustat subgroup. Conversely, the daprodustat subgroup showed a non-significant increase in the change in mean iron. Variations among study groups could not be mitigated through a sensitivity analysis.

Secondary Safety Outcomes

The secondary safety outcomes were also evaluated, with their respective effect sizes, RR, 95% CI, P-values, and I² values presented in Table 6. The various safety outcomes we encountered included the occurrence of any adverse events, any serious adverse events, any drug-related adverse events, drug-related serious adverse events, adverse events leading to withdrawal, any drug-related adverse events leading to death, treatment-emergent adverse events leading to death, 3-4-5 point MACE, hyperkalemia, nausea, vomiting, back pain, pneumonia, hypotension, nasopharyngitis, retinal changes, progression of CKD, and muscle spasm.

**Table 6:** Secondary safety outcomes. The above table shows that apart from an increased incidence of adverse events leading to withdrawal and a higher incidence of diarrhea in the HIFI group, most secondary safety outcomes showed no significant differences between the two drug classes. RR: Relative risk. CI: Confidence interval. I2: Heterogeneity. AEs: Adverse events. TEAE: Treatment emergent adverse events. MACE: Major adverse cardiovascular events. CKD: Chronic kidney disease.

Safety Outcomes	Effect Size (RR)	95%CI	P value	I2
Any AEs	1.01	0.99, 1.02	0.55	31%
Any Serious AEs	1.05	0.99, 1.11	0.08	51%
Any Drug Related Serious AEs	1.06	0.93, 1.20	0.41	0%
AEs Leading to Withdrawal	2.03	1.50, 2.74	0.00001	54%
TEAE Leading to Death	0.98	0.86, 1.11	0.72	0%
Drug Related AEs	1.42	0.54, 3.71	0.47	96%
Drug Related AEs Leading to Withdrawal	4.51	1.53, 13.34	0.47	96%
MACE (3 Point)	1.01	0.94, 1.09	0.79	23%
MACE (4 Point )	0.97	0.85,1.11	0.63	66%
MACE (5 Point)	1.43	0.97, 2.09	0.07	96%
Hypertension	0.94	0.87, 1.01	0.08	4%
Hyperkalaemia	0.93	0.69, 1.27	0.67	30%
Nausea	1.23	0.85, 1.77	0.27	66%
Vomiting	1.11	0.86, 1.42	0.43	36%
Back Pain	0.92	0.66, 1.30	0.65	30%
Diarrhoea	1.3	1.11, 1.51	0.001	21%
Pneumonia	1.08	0.96, 1.22	0.2	0%
Cancer Related Deaths	1.14	0.83, 1.56	0.43	18%
Hypotension	1.08	0.92, 1.26	0.34	3%
Nasopharyngitis	1.01	0.89, 1.15	0.85	29%
Progression of CKD	1.02	0.83, 1.24	0.87	41%
Stroke	1.03	0.39, 2.73	0.96	8%
Retinal Changes	1.13	0.89, 1.44	0.31	0%
Muscle Spasm	0.75	0.42, 1.36	0.35	64%

Any adverse events (%): There was no significant difference association between HIF-PHIs and ESAs in the incidence of any adverse events reported from 16 studies. Any serious adverse events (%): The occurrence of any serious adverse events was slightly higher in the HIF-PHI group than in the ESA group from 14 studies, but this difference was not statistically significant. Any drug-related adverse events (%): As reported from three studies, there was a non-significant slight increased risk of any drug-related adverse events. Owing to the greater heterogeneity, we performed the sensitivity analysis by excluding the Coyne DW et al. study, which decreased the variations among different studies and was statistically significant [18]. Any drug-related serious adverse events (%): A slight non-significant difference was detected in the incidence of any drug-related serious adverse events between the two drug classes among five studies. Adverse events leading to withdrawal (%): Adverse events leading to withdrawal were significantly increased in the HIF-PHI group compared to the ESA group in eight studies. Any drug-related adverse events (%): Any drug-related adverse events leading to withdrawal were increased in HIF-PHI in three studies, but the association was statistically non-significant. TEAE adverse events leading to death (%): Treatment-emergent adverse events leading to death were slightly decreased in nine studies taking HIF-PHI, and was a statistically non-significant association. 3-4-5 point MACE (%): 3-point MACE and 4-point MACE from eight and two studies showed non-significant no association in HIF-PHI compared to ESAs. However, 5-point MACE from six studies showed a non-significant slight increase in risk in HIF-PHI with higher heterogeneity %. After applying sensitivity analysis, the variation among the studies decreased by excluding the Barratt J et al. study [16]. Other side effects (%): No significant differences were seen between the two drug classes for the incidence of hyperkalemia, hypertension, nausea, vomiting, back pain, pneumonia, hypotension, nasopharyngitis, retinal changes, progression of CKD, and muscle spasm from 11, 15, 10, 11, six, eleven, eight, seven, five, four and seven studies, respectively. However, the HIF-PHI group had a significantly higher incidence of diarrhea than the ESA group from 12 studies. In summary, for secondary outcomes, apart from an increased incidence of adverse events leading to withdrawal and a higher incidence of diarrhea in the HIF-PHI group, most secondary safety outcomes showed no significant differences between the two drug classes.

Discussion

Numerous studies have demonstrated the efficacy and safety of ESA used in treating anemia in CKD patients. Anemia in CKD is multifactorial in etiology, but the most widely accepted cause is decreased erythropoietin production by the kidney. This systematic review and meta-analysis included 19 studies with a total of 22,151 patients, examining the effectiveness of HIF-PHI (roxadustat, daprodustat, and vadadustat) compared to ESAs in treating anemia. Anemia is common in older individuals due to age-related physiological mechanisms [26,27]. In the presence of sufficient oxygen, HIF alpha is degraded by prolyl hydroxylase enzymes (PHD), preventing EPO production [28]. However, HIF-PHI stabilizes HIF alpha, leading to increased EPO production and subsequent stimulation of RBC production [29]. This mechanism helps to combat anemia in renal patients. In CKD patients, iron absorption and mobilization dysregulation contribute to renal anemia. Elevated serum hepcidin levels, resulting from decreased GFR and subclinical inflammation, disrupt iron uptake and mobilization, hindering RBC production [30]. Systemic activation of HIF suppresses hepcidin production, promoting iron uptake and utilization. Therefore, HIF-PHI offers a unique benefit by increasing physiological EPO expression while enhancing iron utilization. A study with 13,146 patients revealed that HIF-PHI was effective and well-tolerated in treating anemia of CKD [31]. HIF-PHI demonstrated long-term efficacy in improving Hb levels, with a higher likelihood of reaching target levels than ESAs. HIF-PHI significantly increased Hb levels in comparison with placebo (weighted mean difference (WMD) 1.53, 95% CI: 1.39-1.67) or ESAs (WMD: 0.13, 95% CI: 0.03-0.22). Hepcidin, ferritin, and serum iron levels were decreased, while TIBC and transferrin levels were increased in the HIF-PHI group versus those in the placebo or ESAs group. Non-dialysis-dependent patients benefited from HIF-PHI therapy, but excessive iron consumption increased the risk of iron deficiency, warranting long-term iron supplementation [32]. Thus, more interventional studies are required to fully comprehend the efficacy of HIF-PHI in different subgroups.

The primary outcomes of the study were focused on Hb level change and Hb response. The current standard care for renal anemia involves using EPO and its analogs, supplemented with oral or IV iron administration. However, this approach presents clinical challenges and safety concerns, including hypo responsiveness to EPO, potential adverse effects such as hypertension and cardiovascular events, and the risk of iron overload, which warrants an in-depth study on novel drugs like HIF-PHI [33]. Our meta-analysis is one of the most comprehensive studies comparing HIF-PHI to ESAs in patients with anemia. Importantly, it evaluates three key HIF-PHI: roxadustat, daprodustat, and vadadustat. Our study findings indicated a significant increase in mean Hb levels for patients on HIF-PHI compared to those on ESAs, aligning with previous individual studies and least side effects [7,17,34]. However, the difference in Hb response rate was not significant, suggesting that both treatments are equally capable of inducing a response, which mirrors previous findings [16,35]. Our results showed that roxadustat significantly improved both mean Hb levels and Hb response rate, consistent with recent trials [7,34]. Such discrepancies could be due to differences in study design, sample size, or patient characteristics. ESAs are usually administered along with iron supplementation and may lead to iron overload and dysregulation. However, HIF-PHI groups showed a significant decrease in ferritin values, indicating a potential impact on iron stores. However, it is essential to note that this reduction does not imply iron deficiency, as ferritin levels are influenced by the inflammatory state associated with CKD. Hepcidin, a key regulator of iron absorption and release, is elevated in CKD due to iron overload and inflammation. HIF-PHI treatment effectively reduced hepcidin levels, addressing functional iron deficiency and enhancing iron utilization [36-37]. This hepcidin regulation improves erythropoiesis and reduces inflammation in patients with CKD [38]. This meta-analysis suggests that HIF-PHIs correct anemia by reducing hepcidin levels and enhancing iron transport and utilization [35]. Unlike ESAs that require iron supplementation, HIF-PHIs increase transferrin and TIBC and decrease TSAT, indicating improved iron metabolism [39-41]. Elevated TIBC potentially enhances iron transport and utilization, thereby improving Hb levels [42]. Additionally, HIF inhibitors significantly reduced monthly IV iron use compared to ESAs, suggesting their potential to reduce the need for supplemental iron therapy [43-44]. This could have significant implications, including cost reduction and decreased risk of iron overload complications [45]. These findings highlight the potential benefits of HIF inhibitors in improving anemia management and warrant further investigation [46]. Our analysis also documented a significant increase in mean TIBC with HIF inhibitors [7]. Elevated TIBC potentially enhances iron transport and utilization, thereby improving Hb levels [47-49]. In line with this, our study noted a significant reduction in mean monthly IV iron use with HIF inhibitors compared to ESAs [16, 34]. This finding implies that HIF inhibitors, due to their ability to increase endogenous erythropoiesis and iron utilization, could diminish the need for supplemental iron therapy, reducing healthcare costs and potential complications of iron overload [50].

Given the substantial heterogeneity observed across studies, our analysis offers a unique perspective on navigating this diversity. This heterogeneity could arise from variations in patient populations, dosing regimens, and study designs [51]. Our meta-analysis, comprising 19 studies with 22,151 patients, provides more robust findings due to its larger sample size and focus on the individual effects of different HIFs. Our study boasts several strengths; our meta-analysis incorporates 19 individual studies with a total sample size of 22,151 subjects, giving us adequate power for extrapolating accurate and appropriate results. Employing Egger's and Begg's tests, our paper had no issues with any publication bias. Subgrouping of HIF-PHI drugs could differentiate the individual safety and efficacy properties of the drugs, which no other meta-analysis has attempted successfully. Our study included 33 outcomes with eight outcomes sub-grouped, which helped us to delineate the most effective and safer drug in each outcome, including the rigorous methodology adhering to PRISMA guidelines, the large sample size, and the exclusive focus on RCTs. However, it is essential to acknowledge certain limitations. First, differences in study designs, patient baseline characteristics such as age, sample size, different racial ethnicities, and differences in trial characteristics could have led to heterogeneity. Second, some studies had a shorter study duration, and some had a longer duration. Short-duration studies limited our ability to comment on these agents' enduring impacts, especially with CKD patients who are hyporesponsive to some erythropoietic agents. Various studies used different doses of drugs at different weeks, and most of them did not include the doses of control groups, which can lead to a certain level of uncertainty. Heterogeneity in the patient population may limit the generalizability of our findings. Future studies should aim to rectify these gaps and provide more nuanced insights. In conclusion, our study underscores the promising potential of HIF inhibitors, particularly roxadustat and daprodustat, as superior alternatives to ESAs for managing anemia, especially in terms of enhancing iron utilization and reducing supplemental iron needs. While further studies are warranted, our findings highlight this class's differential effects and the benefits of a personalized approach.

## Conclusions

Owing to various adverse side effects and emerging drug resistance with ESAs in renal patients, it has become necessary to switch anemia therapy in renal patients towards safer, efficacious drugs like HIF-PHIs. Our findings suggested that HIF-PHIs, especially roxadustat and daprodustat, can increase mean Hb levels, increase Hb response, reduce hepcidin levels, increase TIBC, and decrease mean monthly iron use to provide an overall effective iron utilization. HIF-PHIs also showed fewer side effects, such as diarrhea and any adverse events leading to drug withdrawal, as compared to ESA. Thus, we recommend using HIF-PHIs, especially roxadustat and daprodustat, in treating renal anemia over ESAs, as they have a safer profile and are more efficacious than ESAs. Further large-scale, high-quality RCTs are necessary to confirm these findings and potentially guide the future clinical decision-making process.

## Appendices

Appendix 1 

**Table 7:** Detailed search strategy. CKD: Chronic kidney disease; ESRD: End-stage renal disease; HIF-PHI: Hypoxia-inducible factor-prolyl hydroxylase domain inhibitors; ESA: Erythropoietin-stimulating agents; EPO: Erythropoietin.

Database	Supplemental Search Strategy	Results
PubMed	(((((("anaemia"[All Fields] OR "anemia"[MeSH Terms] OR "anemia"[All Fields] OR "anaemias"[All Fields] OR "anemias"[All Fields]) AND "CKD"[All Fields]) OR ("renal insufficiency, chronic"[MeSH Terms] OR ("renal"[All Fields] AND "insufficiency"[All Fields] AND "chronic"[All Fields]) OR "chronic renal insufficiency"[All Fields] OR ("chronic"[All Fields] AND "kidney"[All Fields] AND "disease"[All Fields]) OR "CKD"[All Fields]) OR ("kidney failure, chronic"[MeSH Terms] OR ("kidney"[All Fields] AND "failure"[All Fields] AND "chronic"[All Fields]) OR "chronic kidney failure"[All Fields] OR "esrd"[All Fields]) OR ("kidney failure, chronic"[MeSH Terms] OR ("kidney"[All Fields] AND "failure"[All Fields] AND "chronic"[All Fields]) OR "chronic kidney failure"[All Fields] OR ("end"[All Fields] AND "stage"[All Fields] AND "renal"[All Fields] AND "disease"[All Fields]) OR "end stage renal disease"[All Fields])) AND "HIFI"[All Fields]) OR (("hypoxia"[MeSH Terms] OR "hypoxia"[All Fields] OR "hypoxia s"[All Fields] OR "hypoxias"[All Fields]) AND ("induce"[All Fields] OR "induced"[All Fields] OR "inducer"[All Fields] OR "inducers"[All Fields] OR "induces"[All Fields] OR "inducibilities"[All Fields] OR "inducibility"[All Fields] OR "inducible"[All Fields] OR "inducing"[All Fields]) AND ("factor"[All Fields] OR "factor s"[All Fields] OR "factors"[All Fields]) AND ("antagonists and inhibitors"[MeSH Subheading] OR ("antagonists"[All Fields] AND "inhibitors"[All Fields]) OR "antagonists and inhibitors"[All Fields] OR "inhibitors"[All Fields] OR "inhibitor"[All Fields] OR "inhibitor s"[All Fields]))) AND "ESA"[All Fields]) OR (("EPO"[MeSH Terms] OR "EPO"[All Fields] OR "epoetin alfa"[MeSH Terms] OR ("epoetin"[All Fields] AND "alfa"[All Fields]) OR "epoetin alfa"[All Fields] OR "EPOs"[All Fields] OR "EPO s"[All Fields]) AND ("stimulate"[All Fields] OR "stimulated"[All Fields] OR "stimulates"[All Fields] OR "stimulating"[All Fields] OR "stimulation"[All Fields] OR "stimulations"[All Fields] OR "stimulative"[All Fields] OR "stimulator"[All Fields] OR "stimulator s"[All Fields] OR "stimulators"[All Fields]) AND ("agent"[All Fields] OR "agents"[All Fields]))	2555
Google Scholar	([ Anaemia] AND [ CKD OR ESRD OR end stage renal disease] AND [ HIFI OR HIF inhibitor] AND [ESA OR EPO stimulating agent])	3329
Embase	([ Anaemia] AND [ CKD OR ESRD OR end stage renal disease] AND [ HIFI OR HIF inhibitor] AND [ ESA OR EPO stimulating agent])	521
